# Clinical predictors for nondiabetic kidney diseases in patients with type 2 diabetes mellitus: a retrospective study from 2017 to 2021

**DOI:** 10.1186/s12902-022-01082-8

**Published:** 2022-06-30

**Authors:** Yong-qin Zeng, Yu-xing Yang, Cheng-jing Guan, Zi-wei Guo, Bo Li, Hai-yan Yu, Rui-xue Chen, Ying-qian Tang, Rui Yan

**Affiliations:** 1 Department of Nephrology, The Affiliated Hospital of Guizhou Medical University, Guiyi Street, Yunyan District, Guiyang, 550004 China; 2 Department of Endocrinology, The Affiliated Hospital of Guizhou Medical University, Guiyang, 550004 China

**Keywords:** Nondiabetic kidney disease, Type 2 diabetes mellitus, Diabetic retinopathy, Red blood cell, Diabetes duration, Urinary glucose

## Abstract

**Background:**

Nondiabetic kidney disease (NDKD), which is prevalent among patients with diabetes mellitus (DM), is considerably different from diabetic kidney disease (DKD) in terms of the pathological features, treatment strategy and prognosis. Although renal biopsy is the current gold-standard diagnostic method, it cannot be routinely performed due to a range of risks. The aim of this study was to explore the predictors for differentiating NDKD from DKD to meet the urgent medical needs of patients who cannot afford kidney biopsy.

**Methods:**

This is a retrospective study conducted by reviewing the medical records of patients with type 2 DM who underwent percutaneous renal biopsy at the Affiliated Hospital of Guizhou Medical University between January 2017 and May 2021. The demographic data, clinical data, blood test results, and pathological examination results of the patients were obtained from their medical records. Multivariate regression analysis was performed to evaluate the predictive factors for NDKD.

**Results:**

A total of 244 patients were analyzed. The median age at biopsy was 55 (46, 62) years. Patients diagnosed with true DKD, those diagnosed with NDKD and those diagnosed with NDKD superimposed DKD represented 48.36% (118/244), 45.9% (112/244) and 5.74% (14/244), respectively, of the patient population. Immunoglobulin A nephropathy was the most common type of lesion in those with NDKD (59, 52.68%) and NDKD superimposed DKD (10, 71.43%). Independent predictive indicators for diagnosing NDKD included a DM duration of less than 5 years (odds ratio [OR] = 4.476; 95% confidence interval [CI]: 2.257–8.877; *P* < 0.001), an absence of diabetic retinopathy (OR = 4.174; 95% CI: 2.049–8.502; *P* < 0.001), a high RBC count (OR = 1.901; 95% CI: 1.251–2.889; *P* = 0.003), and a negative of urinary glucose excretion test result (OR = 2.985; 95% CI: 1.474–6.044; *P* = 0.002)..

**Conclusions:**

A DM duration less than 5 years, an absence of retinopathy, a high RBC count and an absence of urinary glucose excretion were independent indicators for the diagnosis of NDKD, suggesting that patients with NDKD may require a different treatment regimen than those with DKD.

## Background

The worldwide prevalence of diabetes mellitus (DM) has increased considerably in the past decades, mainly due to the increased prevalence of type 2 DM (T2DM) [1]. According to data published by the International Diabetes Federation, there are currently 463 million adults with DM (20–79 years) worldwide [2]. Meanwhile, the prevalence rates of macrovascular and microvascular complications associated with DM are also increasing dramatically [1]. Diabetic kidney disease (DKD), a common complication of DM driven by microvascular changes, is the leading cause of end-stage renal disease in developed countries and is a growing epidemic in China [3].

However, DKD is not the only form of kidney disease in patients with DM. Patients diagnosed with chronic kidney disease (CKD) and DM can have true DKD (CKD is a direct consequence of DM, and the histopathological patterns of the kidney are consistent with the changes from DKD, which are characterized by glomerular basement membrane thickening, mesangial matrix expansion, nodular glomerulosclerosis, and arteriolar hyalinosis), nondiabetic kidney disease (NDKD) (CKD caused by nondiabetic factors, such as immunoglobulin A nephropathy [IgA N] and membranous nephropathy [MN]), or a combination of both DKD and NDKD [4]. It has been previously reported that approximately 50% of patients with DM worldwide develop CKD but that only 20–75% of these patients with DM are diagnosed true DKD [5–7].

In clinical practice, the diagnosis of DKD relies predominantly on clinical presentations, including persistent proteinuria, hypertension, and a progressive decline in renal function[8]. Diagnostic kidney biopsy is rarely performed in patients with DM. Naturally, in the absence of a diagnostic biopsy, clinicians often diagnose patients with both DM and CKD as DKD, which inflates the true prevalence of DKD. However, there is considerable heterogeneity in the pathological features and prognoses between DKD and NDKD [4]. It is generally believed that some cases of NDKD are readily treatable or even alleviable, while cases of DKD are not. Therefore, establishing a correct diagnosis in patients with DM superimposed with CKD has decisive influence on the selection of treatment strategies, thus eventually affecting the renal prognosis.

Given that the diabetic environment can alter the progression of NDKD, the form of kidney disease in patients with DM can only be reliably distinguished by performing renal biopsy [9]. However, renal biopsy is an invasive procedure, and it has a range of limitations. First, most clinicians perform renal biopsy in only the patients with DM with indications of NDKD due to the trauma caused by renal biopsy [10]. Second, some patients with DM are unable to tolerate renal biopsy due to them having a poor physical condition. Third, it is difficult to perform renal biopsy at some non-specialist hospitals. Therefore, studies need to be performed to identify the diagnostic predictors for NDKD to meet medical needs for patients with DM who cannot afford kidney biopsy.

## Methods

### Ethical approval

Our study protocol was conducted in accordance with the Declaration of Helsinki guidelines. Given this is a retrospective cohort study, consent for conducting present study was not obtained, but written consent for renal biopsy was obtained from all of the included patients.

### Population and study design

This study was retrospectively conducted by reviewing the medical records of patients with T2DM who underwent percutaneous renal biopsy at the Affiliated Hospital of Guizhou Medical University between January 2017 and May 2021, and the demographic data, clinical data, blood tests results, and pathological examination results were obtained. The diagnosis of T2DM at our institution complies with the criteria defined by American Diabetes Association [11]. CKD was defined as a decreased kidney function demonstrated by a glomerular filtration rate (GFR) of less than 60 mL/min/1.73 m^2^ or markers of kidney damage (includingalbuminuria, urinary sediment abnormalities, electrolyte abnormalities or other abnormalities due to tubular disorders, histological abnormalities, structural abnormalities detected by imaging, and a history of a kidney transplantation), or both, for a duration of at least 3 months, regardless of the underlying cause [12]. The inclusion criteria were as follows: 1) Chinese ethnicity; 2) diagnosed with T2DM at the time of renal biopsy; 3) no previous renal replacement therapy (i.e., hemodialysis, peritoneal dialysis, or kidney transplantation). The criteria for renal biopsy used in this study were uniform for all of the patient. we recommended a renal biopsy pathological examination for a clear diagnosis after excluding contraindications for renal biopsy (i.e., uncorrectable bleeding tendency, severe hypertension, and active kidney infection) to all of the patients.

We first collected demographic information, including age, gender, body mass index ([BMI], calculated by dividing the weight by the height squared, kg/m^2^), smoking status, drinking status, hypertension status (defined as systolic blood pressure ≥ 140 mmHg, diastolic blood pressure ≥ 90 mmHg, or the use of antihypertensive medications)[13], dyslipidemia status (defined as cholesterol ≥ 5.2 mmol/L, triglycerides ≥ 1.7 mmol/L, low-density lipoprotein cholesterol ≥ 2.6 mmol/L, high-density lipoprotein cholesterol < 1.0 mmol/L in men and < 1.3 mmol/L in women, or the use of any lipid-lowering medication)[14], diabetic retinopathy status (DR) diagnosed by an ophthalmologist), DM duration, and hypertension duration (defined as the time from the first diagnosis to the time of the renal biopsy) to assess whether such parameters are associated with the renal pathological type.

Laboratory data, including the blood urea nitrogen level, creatinine level, estimated glomerular filtration rate (eGFR), uric acid level, random blood glucose level, hemoglobin A1c (HbA1c) level, red blood cell (RBC) count, hemoglobin (Hb) level, albumin (Alb) level, urinary glucose excretion, urinary protein level, urinary occult blood, and urinary protein excretion (UPE) obtained over 24 h; data from tumor marker analyses (CA199, CA153, CA125, alpha fetoprotein, carcinoembryonic antigen, etc.); and data from chest computed tomography, abdominal color doppler ultrasound, and other examinations, were collected. Approximately 9% of the patients accepted the detection of anti-phospholipase A2 receptor antibodies for diagnosing MN.

Finally, light microscopy, immunofluorescence, and electron microscopy were performed on all of the biopsy specimens, which were diagnosed by the same group of pathologists. Clinical pathology discussion was conducted by the same group of experienced nephrologists. The pathological diagnosis and classification of DKD were based on the pathological criteria published by the Renal Pathology Society in 2010 [15]. According to the pathological results of the renal biopsy, patients were classified into either the isolated DKD group, isolated NDKD group, or MIX group (NDKD superimposed on DKD).

### Statistical analysis

For continuous variables, the data are presented as the median and interquartile range [median (25th percentile, 75th percentile)] for skewed data and as the mean ± standard deviation for normally distributed data. For categorical variables, the data are presented as the frequency (%). Analysis of variance or the Kruskal–Wallis test was used to compare continuous variables. The chi-square (χ2) test was used to assess the differences between the categorical variables. Univariate and multivariate logistic regression analyses were used to screen for the factors (including demographic information, such as gender, age, and BMI, and the covariates with a *p*-value of < 0.05 in univariate analysis) that were associated with the diagnosis of NDKD A two-sided *p*-value of < 0.05 was considered to indicate statistical significance. The statistical analysis was performed using SPSS software, version 26.0 (IBM Corp., Armonk, NY, USA).

## Results

### Characteristics of the study population

A total of 244 patients were analyzed, 66.66% of whom were male. The median age of the patients at the time of the renal biopsy was 55 (46, 62) years, the median DM duration was 5 (1, 10) years, the median HbA1c was 7% (6.13%, 8.1%), the median eGFR was 42.45 (21.46, 69.64) mL/min/1.73 m^2^, and the median UPE was 3.69 (1.69, 5.89) g/24 h. The numbers of patients with hypertension, dyslipidemia, anemia and DR were 155 (63.52%), 213 (87.30%), 155 (63.52%), and 96 (39.34%), respectively. The patients were divided into three according to the type of renal pathology: the DKD (118, 48.36%), NDKD (112, 45.90%), and MIX (14, 5.74%) groups. The baseline clinical characteristics of the three groups are shown in Table 1. There was no significant difference in the age, gender, BMI, smoking and drinking statuses, hypertension duration, and dyslipidemia status of patients among three groups. The DM duration was the longest in the MIX group, followed by the DKD group. It was the shortest in the NDKD group. Compared with the patients in the DKD and MIX groups, the patients in the NDKD group had lower incidence rates of hypertension, DR, and urinary glucose and lower levels of UPE, but they had a higher eGFR, higher RBC count, higher Hb level, and higher urinary RBC count.

**Table 1:** Baseline clinical and biochemical characteristics of the study population in the classification group

parameters	DKD Group (*n* = 118)	NDKD Group (*n* = 112)	MIX Group (*n* = 14)	*P* value
Male, n(%)	71.00 (60.17)	69.00 (61.61)	8.00 (57.14)	0.939
Age, years, n(%)	54.50 (48.00, 61.00)	54.50 (42.25, 62.00)	61.50 (50.25, 65.00)	0.251
BMI, kg/m^2^	25.06 ± 3.41	25.59 ± 4.55	24.53 ± 2.40	0.383
Tobacco smoking, n(%)				0.471
Yes	47.00 (39.83)	37.00 (31.04)	4.00 (28.57)	
No	71.00 (60.17)	75.00 (66.96)	10.00 (71.43)	
Alcohol drinking, n(%)				0.575
Yes	38.00 (32.20)	29.00 (25.89)	4.00 (28.57)	
No	80.00 (67.80)	83.00 (74.11)	10.00 (71.43)	
Diabetes duration, years, n(%)				< 0.001
< 5	28.00 (23.73)	74.00 (66.07)	3.00 (21.43)	
≥ 5	90.00 (76.27)	39.00 (34.83)	11.00 (78.57)	
DR, n(%)				< 0.001
Yes	67.00 (56.78)	23.00 (20.54)	6.00 (42.86)	
No	51.00 (43.22)	89.00 (79.46)	8.00 (57.14)	
Hypertension, n(%)				0.008
Yes	87.00 (73.73)	62.00 (55.36)	11.00 (78.57)	
No	31.00 (26.27)	50.00 (44.64)	3.00 (21.43)	
Hypertension duration, years, n(%)				0.729
< 5	90.00 (76.27)	90.00 (80.36)	10.00 (71.43)	
≥ 5	28.00 (23.73)	22.00 (19.64)	4.00 (28.57)	
dyslipidemia				0.300
Yes	102.00 (86.44)	101.00 (90.18)	11.00 (78.57)	
No	16.00 (13.56)	11.00 (9.82)	3.00 (21.43)	
Serum calcium, mg/dL	2.07 (1.97, 2.19)	2.15 (1.99, 2.28)	2.06 (1.97, 2.18)	0.048
BUN, mmol/L	9.45 (6.80, 12.98)	8.10 (5.62, 12.21)	10.45 (7.88, 19.79)	0.078
Cr, μmol/L	157.85 (112.03, 241.31)	128.86 (78.55, 240.98)	202.52 (169.32, 309.77)	0.006
eGFR, mL/min/1.73m^2^	37.98 (20.92, 58.49)	52.86 (21.81, 78.98)	23.56 (15.70,32.25)	0.003
Uric acid, μmol/L	391.85 (320.85, 456.88)	421.30 (336.50, 510.13)	376.90 (358.90, 445.28)	0.134
RBG, mmol/L	9.50 (7.06, 12.68)	7.62 (5.93, 10.82)	11.05 (5.73, 13.74)	0.029
HbA1c, %	7.13 (6.18, 8.96)	6.80 (6.10, 7.90)	6.87 (5.83, 7.90)	0.463
RBC count, × 10^12^/L	3.67 (3.16, 4.21)	4.30 (3.79, 5.00)	3.33 (3.05, 3.77)	< 0.001
Hb, g/L	107.00 (91.50, 125.00)	125.00 (113.25, 147.75)	96.00 (90.00, 113.25)	< 0.001
Albumin, g/L	32.92 (28.20, 38.50)	34.10 (28.16, 43.32)	31.89 (29.60, 39.30)	0.325
UPE, g/24 h, n(%)				0.006
< 3.5	48.00 (40.68)	62.00 (55.36)	6.00 (42.86)	
≥ 3.5	70.00 (59.32)	50.00 (44.64)	8.00 (57.14)	
Glucose in urine, n(%)				< 0.001
Positive	62.00 (52.54)	30.00 (26.79)	6.00 (42.86)	
Negative	56.00 (47.46)	82.00 (73.21)	8.00 (57.14)	
Occult blood in urine, n(%)				0.495
Positive	65.00 (55.08)	66.00 (58.93)	6.00 (42.86)	
Negative	53.00 (44.92)	46.00 (41.07)	8.00 (57.14)	
RBC count in urine, count/HP				0.260
	5.00 (2.00, 14.00)	6.00 (0.00, 33.00)	1.50 (0.00, 8.75)	

The data were presented as median and interquartile range [median (25th percentile, 75th percentile)] and mean ± standard deviation for skewed data and normally distributed data, respectively; categorical data were presented as the frequency (%). Kruskal–Wallis test and the chi-square test were used to compare continuous variables and categorical variables, respectively. *BMI* Body mass index, *DR* Diabetic retinopathy, *BUN* Blood urea nitrogen, *Cr* Serum creatinine, *eGFR* Estimated glomerular filtration rate, *RBG* Random blood glucose, *HbA1c* Hemoglobin A1c, *RBC* Red blood cell, *Hb* Hemoglobin, *UPE* Urinary protein excretion

It is noteworthy that up to 87.29% of patients in the DKD group had class III or IV of diabetic nephropathy at the time of renal biopsy, while 100% of the patients in the MIX group had the same (Table 2). There were 14 pathological types in the NDKD group, including primary and secondary glomerular diseases. IgA N was the most common type of lesion, accounting for 52.68% (59/112) of all lesions in the patients with NDKD, and the second-most common type was MN, accounting for 18.75% (21/112) of all lesions in the patients with NDKD. The other 32 patients were diagnosed with other renal diseases (Table 3). Similar to the results in the NDKD group, IgA N (10, 71.43%) and MN (2, 14.29%) were the most common and second-most common types, respectively, in the MIX group.

**Table 2:** The glomerular classification of diabetic kidney disease

	All (*n* = 244)	DKD Group (*n* = 118)	MIX Group (*n* = 14)
Class I, n(%)	3 (1.23)	3 (2.54)	0
Class IIa, n(%)	2 (0.82)	2 (1.69)	0
Class IIb, n(%)	10 (4.10)	10 (8.48)	0
Class III, n(%)	95 (38.93)	84 (71.19)	11 (78.57)
Class IV, n(%)	22 (9.02)	19 (16.10)	3 (21.43)

The data were presented as the frequency (%). NDKD, diabetic kidney disease; MIX Group, NDKD superimposed on DKD

**Table 3:** Pathological types in Non-diabetic kidney disease

Type	All (*n* = 244)	NDKD Group (*n* = 112)	MIX Group (*n* = 14)
IgA N, n( %)	69 (28.28)	59 (52.68)	10 (71.43)
MN, n( %)	23 (9.43)	21 (18.75)	2 (14.29)
MCD, n( %)	6 (2.46)	6 (5.36)	0
MsPGN, n( %)	5 (2.05)	5 (4.46)	0
Hypertensive nephropathy, n( %)	5 (2.05)	4 (3.57)	1 (7.14)
HSPN, n( %)	5 (2.05)	5 (4.46)	0
PsG, n( %)	3 (1.23)	3 (2.68)	0
Amyloid nephropathy, n( %)	4 (1.64)	3 (2.68)	1 (7.14)
ANCA relative glomerulonephritis, n( %)	4 (1.64)	4 (3.57)	0
FSGS, n( %)	3 (1.23)	3 (2.68)	0
LN, n( %)	3 (1.23)	3 (2.68)	0
MPGN, n( %)	1 (0.41)	1 (0.89)	0
C3G, n( %)	1 (0.41)	1 (0.89)	0
OaG, n( %)	1 (0.41)	1 (0.89)	0

The data were presented as the frequency (%). *NDKD* Non-diabetic kidney disease; MIX Group, NDKD superimposed DKD, *IgA N* IgA nephropathy, *MN* Membranous nephropathy, *MCD* Minimal change disease, *MsPGN* Mesangial proliferative glomerulonephritis, *HSPN* Henoch-Schonlein purpura nephritis, *PsG* Proliferative-sclerosing glomerulonephritis, *FSGS* Focal segmental glomerulosclerosis, *LN* Lupus nephritis, *MPGN* Membranoproliferative glomerulonephritis, *C3G* C3 glomerulopathy, *OaG* Obesity-associated glomerulomegaly. Six patients were diagnosed as IgA N combined with MN, and one patients was diagnosed as MCD combined with hypertensive nephropathy

### Factors associated with the diagnosis of NDKD

Given the small number of patients in the MIX group which may imply a limited predictive value, we excluded these patients from further analysis. The univariate regression analysis identified a number of parameters that were significantly correlated with the diagnosis of NDKD, including the shorter DM duration (< 5 years) (odds ratio [OR] = 5.030; 95% confidence interval [CI]: 2.868–8.824; *P* < 0.001), absence of hypertension (OR = 2.263; 95%CI: 1.301–3.938; *P* = 0.004), absence of DR (OR = 5.084; 95% CI: 2.831–9.130; *P* < 0.001), eGFR (OR = 1.013; 95% CI: 1.005–1.022; *P* = 0.002), RBC count (OR = 2.494; 95% CI: 1.758–3.537; *P* < 0.001), Hb level (OR = 1.032; 95% CI: 1.019–1.044; *P* < 0.001), negative of urinary glucose status (OR = 3.026; 95% CI: 1.741–5.259; *P* < 0.001), urinary RBC count (OR = 1.008; 95% CI: 1.002–1.014; *P* = 0.008), and reduced UPE level (< 3.5 g/24 h) (OR = 1.857; 95% CI: 1.096–3.145; *P* = 0.021). The demographic data, including gender, age, BMI, and variables that were observed to be statistically significant in the univariate analysis were used to further multivariate logistic regression analysis to identify the factors closely related to NDKD. The results demonstrated that four indicators were statistically significant in the diagnosis of NDKD: a diabetes duration of less than 5 years (OR = 4.476; 95% CI: 2.257–8.877; *P* < 0.001), an absence of DR (OR = 4.174; 95% CI: 2.049–8.502; *P* < 0.001), the RBC count (OR = 1.901; 95% CI: 1.251–2.889; *P* = 0.003), and a negative test result for urinary glucose (OR = 2.985; 95% CI: 1.474–6.044; *P* = 0.002) (Table 4).

**Table 4:** Multivariate regression analysis of factors related to the diagnosis of NDKD

parameters	B	OR	95%CI	*P* value
Shorter Diabetes duration (< 5 years)	1.499	4.476	2.257–8.877	< 0.001
Absence of DR	1.429	4.174	2.049–8.502	< 0.001
RBC count	0.643	1.901	1.251–2.889	0.003
Negative of urinary glucose	1.094	2.985	1.474–6.044	0.002

*DR* Diabetic retinopathy, *RBC* Red blood cell

## Discussion

With the widespread implementation of renal biopsy, NDKD is prevalent in the population of patients with DM [6, 16, 17]. It has been realized that not all patients with DM diagnosed with CKD develop renal dysfunction as a consequence of DM. Importantly, DKD and NDKD have considerably different treatment strategies and prognoses. Renal biopsy remains the only reliable way to distinguish the type of kidney disease in patients with DM. However, as an invasive procedure, renal biopsy has a range of limitations [10]. Therefore, there is an urgent need for clinicians to identify reliable predictors to be able to accurately distinguish diabetic patients with NDKD from DKD to meet the urgent medical needs of patients who cannot afford renal biopsy. In our study, 244 patients diagnosed with T2DM at the time of biopsy were analyzed to screen for the predictive factors of NDKD in patients with T2DM.

Some studies have investigated the prevalence of NDKD in cohorts of patients with DM and observed that the prevalence ranged from 18 to 62% [6, 17, 18]. In this study, 45.90% (112/244) of patients were diagnosed as isolated NDKD based on renal biopsy pathological results, which is a result that is consistent with previous studies. Furthermore, 5.74% (14/244) of patients were diagnosed as both DKD and NDKD. These results suggest that clinicians should pay more attention to the possibility of NDKD developing in patients with T2DM. Fourteen pathological types of NDKD were observed in the present study. For both the NDKD group and MIX group, IgA N was the most common type of lesion (52.68% and 71.43% in the NDKD group and MIX group, respectively), followed by MN (18.75% and 14.29% in the NDKD group and MIX group, respectively). This result is consistent with studies conducted Zhuo L, et al. and Fiorentino M, et al. [19, 20], but different with those of Liu D [21], which showed MN was the most common type of NDKD in patients with DM. This difference may be caused by different renal biopsy criterion. We recommended that all patients with CKD superimposed on T2DM, undergo renal biopsy unless contraindications exist, whereas they mainly detected patients with nephrotic syndrome. For both the DKD group and MIX group, a high percentage of patients have reached class III or IV of diabetic nephropathy at the time of renal biopsy (87.20% and 100.00% in the NDKD group and MIX group, respectively), which maybe due to the lack of symptoms in early diabetic nephropathy then delayed the patients’ visit. Therefore, it is necessary to conduct regular urine tests and other screenings for kidney diseases in diabetic patients.

Further analysis showed that compared with DKD and MIX group, patients in the NDKD group had a lower incidence of hypertension, DR and urinary glucose, reduced UPE level, shorter DM duration, higher eGFR level, RBC count, Hb level and urinary RBC count. Multivariate logistic regression analysis showed that DM duration less than 5 years, absence of DR and higher RBC count were significant and independent predictive factors for diagnosis of NDKD. It is generally accepted that the risk of nephropathy increases with prolonged DM duration. Previous studies have proposed that DM duration is closely related to the prevalence of nephropathy in patients with T1DM [22]. Unlike T1DM, it is difficult to define the onset of T2DM accurately due to occult symptoms in the early stage of T2DM and lack of awareness and regular physical examination about this disease in most people. But several studies still showed that DM duration was shorter in diabetic patients with NDKD than DKD [5, 23], our results support this finding once again.

Both DR and DKD are common microvascular complication of T2DM. It is generally believed that the development of DR is often accompanied by that of DKD[24], so the patients without DR are more likely to have NDKD. Previous studies have shown that the incidence of DR in patients with NDKD ranged from 4.7%–14.9% [17, 18, 25, 26]. In the present study, the incidence of DR in the NDKD group was 20.54%, a bit higher than the previous data. The reason may be that we conducted ophthalmoscope examination for all patients with T2DM, thus avoiding the possibility of missing a diagnosis. Our data revealed that the absence of DR could be a factor for predicting NDKD in patients with T2DM, but it is not an exclusion criterion for DKD. Notably, a cohort study proposed that diabetic nephropathy was also an independent risk factor for the development and progression of DR [27]. In one word, our results reminds nephrologists again that routine ophthalmoscope examination should be performed on patients with T2DM to diagnose and treat early.

To our knowledge, the present study suggested that higher RBC count and negative of urinary glucose had predictive value for diagnosis of NDKD for the first time. Previous studies have explored the level of Hb not RBC count in patients with NDKD and DKD, and suggested that higher level of Hb is a predictive factor for NDKD [28, 29]. In our study, univariate regression analysis showed that both RBC count (OR = 2.494; 95% CI: 1.758–3.537; *P* < 0.001) and Hb level (OR = 1.032; 95% CI: 1.019–1.044; *P* < 0.001) are associated with NDKD, but after adjusting gender, age and BMI, et, multivariate logistic regression analysis showed that RBC count (OR: 1.901; 95% CI: 1.251–2.889; *P* = 0.003) not Hb level has a predictive value for the diagnosis of NDKD. It makes sense, synthesizing erythropoietin (EPO) is one of the key duty of kidney [30]. Renal anemia is common in CKD patients and also one of the most common complications of DKD, which mainly related to the deficiency of EPO [31]. A study reported that EPO-related anemia can occur early in patients with DKD, but usually does not occur in patients with NDKD of similar severity [32]. One possible underlying mechanism is that early lesions in patients with DKD mainly involve diffuse thickening of the glomerular basement membrane and mesangial matrix hyperplasia [15], which could greatly affect the synthesis of EPO, but NDKD does not. Given EPO is the major stimulator of RBC production [33], it is not surprising that RBC count is more predictive than Hb level. Furthermore, it is also the first time that urinary glucose was collected in predictive analysis for the diagnosis of NDKD. Although there was no significant difference of HbA1c level between patients with DKD and NDKD, patients in DKD groups had higher level of random blood glucose, which may contribute to positive of urinary glucose in patients with DKD. Other underlying pathogenesis requires further exploration.

As for other parameters, there is controversy of the clinical value of UPE between patients with DKD and NDKD [18, 34]. The results of the present study showed that the UPE level was higher in the DKD group versus the NDKD group, but it is not associated with the diagnosis of NDKD. These differences may be related to the criterion of renal biopsy and region, et. We also found that the eGFR in the NDKD group was higher than that in the DKD group, but it is not associated with the diagnosis of NDKD. As a chronic complication of DM, DKD has a slowly onset, the lack of obvious symptoms usually bring a long duration of disease and significant damage to kidney function. However, most patients with NDKD such as IgA N and MN with an acute onset are more likely to show obvious symptoms such as proteinuria and edema, thus can seek hospital visit early.

Although many studies have explored the differential diagnosis of DKD and NDKD, the results from these studies varied, thus differentiating NDKD from DKD effectively and based on scientific evidence remains under further exploration. A strength of this study is that we recommended to all of the patients to undergo renal biopsy unless there were contraindications for it, which minimizes the selection bias as much as possible. In addition, we included as many relevant parameters as possible. However, there are also some limitations to this study. This study is retrospective and was performed at a single center, so applying the results to populations in other countries and regions will produce results that need to be interpreted cautiously due to the restrictive patient population in this study; Moreover, it cannot be entirely ruled out that there were no unmeasured confounding factors that could have possibly influenced the observed association in this study.

## Conclusions

Diabetic nephropathy is regarded as a major cause of end-stage renal disease, and true DKD usually requires treatment by glycaemic control and dual sodium-dependent glucose transporters 2 (SGLT2) and rennin angiotensin system (RAS) inhibition. However, NDKD may require additional therapeutic approaches. The present study demonstrated that a DM duration of less than 5 years, the absence of retinopathy, an elevated of RBC count, and the absence of urinary glucose excretion are independent indicators associated with the diagnosis of NDKD, suggesting that patients with NDKD may require a different treatment regimen than those with DKD when kidney biopsy cannot be performed. Given this study was performed at a single center, the conclusions derived from this work should be further analyzed and validated at other centers in China.

## Data Availability

The datasets generated and/or analysed during the current study are not publicly available due to relevant data protection but are available from the corresponding author on reasonable request.

## Abbreviations

Alb: Albumin
BMI: Body mass index
BUN: Blood urea nitrogen
CI: Confidence interval
CKD: Chronic kidney disease
Cr: Creatinine
DR: Diabetic retinopathy
DM: Diabetes mellitus
DKD: Diabetic kidney disease
eGFR: Estimated glomerular filtration rate
EPO: Erythropoietin
Hb: Hemoglobin
HbA1c: Hemoglobin A1c
IgA N: IgA nephropathy
MN: Menbrane nephropathy
NDKD: Non-diabetic kidney disease
OR: Odds ratio
RBC: Red blood cell
T2DM: Type 2 diabetes mellitus
UPE: Urinary protein excretion
